# Scale and pattern of atrophy in the chronic stages of moderate-severe TBI

**DOI:** 10.3389/fnhum.2014.00067

**Published:** 2014-03-31

**Authors:** Robin E. A. Green, Brenda Colella, Jerome J. Maller, Mark Bayley, Joanna Glazer, David J. Mikulis

**Affiliations:** ^1^Cognitive Neurorehabilitation Sciences Laboratory, Research Department, Toronto Rehabilitation InstituteToronto, ON, Canada; ^2^Department of Psychiatry, Faculty of Medicine, University of TorontoToronto, ON, Canada; ^3^Brain Stimulation and Neuroimaging Laboratory, Monash Alfred Psychiatry Research Centre, Alfred HospitalMelbourne, VIC, Australia; ^4^fMRI Laboratory, Division of Applied and Interventional Research, Toronto Western Research InstituteToronto, ON, Canada; ^5^Department of Medical Imaging, Faculty of Medicine, University of TorontoToronto, ON, Canada

**Keywords:** atrophy, chronic, degeneration, traumatic brain injury, progression, MRI

## Abstract

**Background:** Moderate-severe traumatic brain injury (TBI) is increasingly being understood as a progressive disorder, with growing evidence of reduced brain volume and white matter (WM) integrity as well as lesion expansion in the chronic phases of injury. The scale of these losses has yet to be investigated, and pattern of change across structures has received limited attention.

**Objectives:** (1) To measure the percentage of patients in our TBI sample showing atrophy from 5 to 20 months post-injury in the whole brain and in structures with known vulnerability to acute TBI, and (2) To examine relative vulnerability and patterns of volume loss across structures.

**Methods:** Fifty-six TBI patients [complicated mild to severe, with mean Glasgow Coma Scale (GCS) in severe range] underwent MRI at, on average, 5 and 20 months post-injury; 12 healthy controls underwent MRI twice, with a mean gap between scans of 25.4 months. Mean monthly percent volume change was computed for whole brain (ventricle-to-brain ratio; VBR), corpus callosum (CC), and right and left hippocampi (HPC).

**Results:** (1) Using a threshold of 2 z-scores below controls, 96% of patients showed atrophy across time points in at least one region; 75% showed atrophy in at least 3 of the 4 regions measured. (2) There were no significant differences in the proportion of patients who showed atrophy across structures. For those showing decline in VBR, there was a significant association with both the CC and the right HPC (*P* < 0.05 for both comparisons). There were also significant associations between those showing decline in (i) right and left HPC (*P* < 0.05); (ii) all combinations of genu, body and splenium of the CC (*P* < 0.05), and (iii) head and tail of the right HPC (*P* < 0.05 all sub-structure comparisons).

**Conclusions:** Atrophy in chronic TBI is robust, and the CC, right HPC and left HPC appear equally vulnerable. Significant associations between the right and left HPC, and within substructures of the CC and right HPC, raise the possibility of common mechanisms for these regions, including transneuronal degeneration. Given the 96% incidence rate of atrophy, a genetic explanation is unlikely to explain all findings. Multiple and possibly synergistic mechanisms may explain findings. Atrophy has been associated with poorer functional outcomes, but recent findings suggest there is potential to offset this. A better, understanding of the underlying mechanisms could permit targeted therapy enabling better long-term outcomes.

## Introduction

Traumatic brain injury (TBI) is increasingly being understood as a chronic and possibly progressive disease, rather than an injury with a circumscribed period of recovery and a static course thereafter (Ng et al., 2008; Masel and Dewitt, 2010; Bigler, 2013a). Evidence is accumulating from several laboratories including our own that following early cognitive recovery, many patients show statistically and clinically significant cognitive decline in the ensuing months (Ruff et al., 1991) and years (Millis et al., 2001; Till et al., 2008). We and others have also demonstrated that the brain's structure is not static after resolution of acute injuries, with TBI patients showing volume loss and reduced white matter (WM) integrity during the sub-acute and chronic stages of injury (Bendlin et al., 2008; Greenberg et al., 2008; Ng et al., 2008; Sidaros et al., 2009; Farbota et al., 2012; Adnan et al., 2013). Given that such atrophy is observed in some studies well after the resolution of acute events (Greenberg et al., 2008; Ng et al., 2008; Ross et al., 2012), and that sub-acute structural deterioration has been correlated with functional and behavioral outcomes (Bendlin et al., 2008; Sidaros et al., 2009), these changes cannot be attributed simply to encephalomalacia (i.e., scar formation associated with gliosis) or to resolution of edema. The evidence is thus supportive of a progressive neuropathology, possible mechanisms of which have been recently reviewed (Smith et al., 2013).

This very different course of TBI than has been traditionally assumed has clinical and scientific implications for how we understand and treat TBI patients, in both the short and longer term. Therefore, it is important to gain an understanding of the scale of atrophy: What proportion of TBI patients demonstrates progressive loss of brain volume? Previous studies have demonstrated group differences in atrophy and/or WM integrity loss between TBI patients and controls. What has yet to be investigated is the actual prevalence of patients who show decline, and moreover, who do so at what might be considered a clinically significant degree. Such information is needed to understand whether it is a subset of TBI patients who are at risk of degeneration or the majority. While atrophy in a subset would direct researchers (and clinicians) to evaluate risk factors for degeneration; ubiquitous atrophy would suggest that TBI itself is a degenerative disorder. The central aim of this study, therefore, was to begin to shed light on this question of scale.

To address the question, we undertook volumetric MRI measurements in TBI patients at approximately 5 and 20 months post-injury and compared the extent of volume loss across time for each patient to a normative control sample, also measured at two time points. We called patients showing significant volume loss “decliners.” In both samples, we examined the whole brain using ventricle-to-brain ratio (VBR), as well as the hippocampi and corpus callosum (CC) and their sub-structures, areas with demonstrated vulnerability to the acute mechanical and neurochemical effects of injury (e.g., axonal deformation, hypoxia, excitotoxic cascades Povlishock and Katz, 2005). Our cohort ranged in severity from complicated mild to severe, but was overall a severely injured group of patients.

With regard to prediction of the scale of atrophy, in a previous study in which we examined lesion expansion, we found that in our sample of 14 patients, 10 showed lesion expansion across time within the chronic stages of injury (Ng et al., 2008). We therefore predicted that at least half of our sample in the current study would show significant volume loss on at least one measure.

We were also interested in the relative vulnerability of the individual structures and sub-structures, and in their pattern of deterioration. With regard to relative vulnerability, we measured the number of decliners (i.e., people who showed atrophy across time points as compared to normative controls) for each structure in order to ascertain whether one structure showed a greater frequency of decliners than another structure.

We speculated that those regions most vulnerable to *acute* injury would show the greatest chronic atrophy. This is because of growing evidence that while “use” increases brain (and in particular hippocampal) volume (Draganski et al., 2006; Maguire et al., 2006), “disuse” mediates volume loss (Underwood and Coulson, 2008; Miller et al., 2013). Therefore, we reasoned that regions most susceptible to initial damage, should sustain greater disuse, and therefore greater later atrophy. Both the CC and the hippocampi are commonly affected in TBI, especially when rotational forces are involved. In one study in children, the hippocampus was identified as the most vulnerable structure to TBI (Wilde et al., 2006), and it is vulnerable to acute phase damage from a variety of mechanisms, (e.g., mechanical deformation, hypoxic/excitotoxic injury, afferent and efferent disconnection of projections, and compromised neurogenesis Greer et al., 2012). The hippocampal head has been shown to be disproportionately vulnerable, as compared to the body and tail (Ariza et al., 2006). We therefore predicted that more individuals would show chronic degeneration in the hippocampal head than any other structure/sub-structure examined.

With regard to the pattern of deterioration, we asked whether the volumes of those regions measured in the study shrink commensurately with one another, and whether their sub-regions would shrink commensurately. In other words, did individuals who showed atrophy on one structure, also necessarily show atrophy on another?

In our previous research into sub-acute cognitive decline (Till et al., 2008), we observed marked variability in the cognitive and psychomotor functions that showed decline from 12 to 24 months post-injury, both within and between subjects. This suggested that atrophy might not have a predictable pattern. On this basis, we predicted that decline in one region would *not* be associated with decline in another, although we expected VBR—as an index that subsumes all other structures—to be associated with the hippocampi and CC.

## Materials and methods

All participants gave their informed consent, except where participants were clinically judged as unable to provide informed consent; here, a substitute decision maker provided informed consent. In such cases, all patients gave their assent to participate. The study was approved by the Research Ethics Board of the Toronto Rehabiltation Institute where the research took place

### Participants

#### TBI patient group

Patient participants were 56 males and females with clinically confirmed TBI. See Table 1 for injury and demographic characteristics of the sample, which were ascertained through medical records, clinical interview and direct testing. Overall, this was a typical group of adult TBI patients with more males than females, more motor-vehicle accidents than other causes of injury, estimated pre-morbid IQ (as measured by the Wechsler Test of Adult Reading (WTAR) Weschler, 2001; Green et al., 2008) in the average range, and just over high-school education. TBI severity for participants was based on the lowest Glasgow Coma Scale (GCS) score obtained from the acute care medical chart, where GCS was available and valid, or from length of post-traumatic amnesia (PTA), which was obtained from medical records and/or from clinical interview with the patient and family members at approximately 2 months post-injury. PTA classifications were based on Lezak (2004). As indicated in Table 1, patients ranged in severity from complicated mild to severe or greater, with mean TBI severity based on GCS in the severe range (*M* = 6.2).

**Table 1:** **Injury and demographic characteristics of TBI sample (*N* = 56)**.

**Variable**	**Proportion/mean**	***SD* (range)**
Age (years)	*M* = 40.16	15.63 (17–73)
Education (years)	*M* = 13.63	3.36 (6–21)
Estimated pre-morbid IQ (WTAR) (*N* = 51)	*M* = 98.45	18.26 (67–125)
Sex	73% = male	
	27% = female	
**SOCIO-ECONOMIC STATUS (BASED ON HOLLINGSHEAD CLASSIFICATION Hollingshead and Redlich, 1958)**		
1. Major business/professional	3.8%	
2. Medium business/minor professional, technical	41.5%	
3. Skilled craftsperson, clerical, sales worker	17%	
4. Machine operator, semiskilled worker	18.9%	
5. Unskilled laborer, menial service worker	18.9%	
**TYPE OF INJURY**		
Motor vehicle accident	57.1%	
Fall	37.5%	
Assault	3.6%	
Sports injury	1.8%	
**SEVERITY OF INJURY VARIABLES**		
Acute care length of stay (days)	*M* = 37.44days	20.01 (5–98)
GCS (lowest of recorded scores)	*M* = 6.19	3.42(3–13)
Mild (13–15)	10.7	
Moderate (9–12)	5.4%	
Severe (≤8)	69.6%	
Missing data	14.3%	
**LENGTH OF POST-TRAUMATIC AMNESIA**		
Less then 5 min, very mild	3.6%	
1–24 h, moderate	1.8%	
1–7 days, severe	21.4%	
1–4 weeks, very severe	44.6%	
>4 weeks, extremely severe	23.2%	
Missing data	5.4%	

All patient participants were recruited at a large, urban rehabilitation teaching hospital in Toronto from the in-patient Acquired Brain Injury service. The patients were part of an ongoing, prospective study on recovery from TBI (*The Toronto Rehab Traumatic Brain Injury Recovery Study*), which includes cognitive, motor, and neuro-imaging assessments at multiple time points. Inclusion criteria for the larger study comprise: clinically confirmed TBI with central (as opposed to orthopedic) injuries severe enough to warrant in-patient neurorehabilitation; out of PTA by 3 months post-injury; aged between 17 and 75; able to use at least one upper extremity; and, functional command of English. An additional inclusion criterion for the present study was completion of two or more MRIs. Exclusion criteria for the larger study include: past history of TBI; history of psychotic or neurological illness; and, TBI sustained secondary to another neurological event (e.g., a stroke). An additional exclusion criterion for the current study was an intervening neurological event between the first and follow-up MRI with the potential to influence the structural status of the brain (e.g., another TBI or intra-cranial infection).

#### Healthy control group

Twelve healthy adult control participants were recruited to the current study. These individuals were students and staff members at the rehabilitation hospital or friends or family members of students and staff members. Controls were excluded if they had a previous history of TBI, including concussion, or any other disease affecting the central nervous system. The control group was 50% male, with a mean age of 36.3 years (*SD* = 12.5, range 18–60), and a mean education of 17.5 years (*SD* = 2.2, range 11–21).

### Design and procedures

The study was a prospective, repeated measures design. The normative control group was used to establish the level of decline in each patient for each structure. This was computed for each subject for each structure, as described below. MRIs were administered at two time points. The first was completed for all patients at a mean of 5.2 months post-injury, (*SD* = 1.15; range: 3.7–10.4). The second scan was at a mean of 20 months post-injury (*SD* = 4.7; range = 10.5–56.1), with a mean gap between scans of 14.8 months (*SD* = 10.9; range = 4.4–52.2). For control participants, the mean gap between scans was 25.4 months (*SD* = 10.0; range = 10.4–39.4). The relatively greater gap in the controls biased hypotheses against Type I errors. The longer gap between control scans allowed for greater non-specific decline to transpire, thereby increasing the threshold for reaching decline in the patients.

The MRI outcome measures were as follows: computed monthly percent change for ventricle to brain ratio (VBR), volumes of the left hippocampus (HPC-L; i.e., HPC-L total, HPC-L head, HPC-L body, HPC-L tail), volumes of the right hippocampus (HPC-R; i.e., HPC-R total, HPC-R head, HPC-R body, HPC-R tail), and CC volumes (i.e., CC total, CC genu, CC body, CC splenium).

All participants were required to pass a rigorous, clinical screening procedure prior to the first MRI assessment. All MRIs were conducted at a separate site (Toronto Western Hospital), which is part of the same center. All equipment and acquisition parameters were identical for the initial and follow-up assessments. One of two MRI technologists performed all MRIs.

#### Acquisition protocol

MRI scans were acquired on a General Electric (GE) Signa-Echospeed 1.5 Tesla HD scanner (SIGNA EXCITE, GE Healthcare, Milwaukee WI), using an eight channel head coil. Sequences included sagittal T1 (*TR*/*TE* = 300/13 ms), slice thickness = 5 mm, space 2.5 mm, matrix 256 × 128 axial gradient recalled echo (GRE) *TR*/*TE* = 450/20, flip angle = 20°, slice thickness = 3 mm no gap, matrix 256 × 192 axial fluid-attenuated-inversion-recovery (FLAIR) *TR*/*TE* = 9000/45 ms, *TI* (inversion time) = 2200 ms, slice thickness = 5 mm no gap, matrix 256 × 192 axial fast spin echo (FSE) proton density (PD)/T2 *TR/TE* 5500/30,90 ms, slice thickness = 3 mm no gap, matrix 256 × 192. All above mentioned sequences were obtained with a 22 cm field of view (FOV). The high-resolution isotropic T1 weighted, three-dimensional IR prepped radio-frequency spoiled-gradient recalled-echo (3D IRSPGR) images *TI*/*TR*/*TE* = 12/300/5,TI, *FA* = 20, slice thickness = 1 mm no gap, matrix = 256 × 256 were acquired in the axial plane utilizing a 25 cm FOV. The entire scanning session lasted approximately 55 min.

#### Image processing and analysis

The MR images were transferred to a workstation for image processing. The scans were received in the Digital Imaging and Communications in Medicine (DICOM) file format and were subsequently converted into (Medical Imaging Network Common Data Form; MINC) file format that was created at McConnell Brain maging Centre of the Montreal Neurological Institute. Following this procedure, the files were anonymized.

A number of image processing steps were performed in order to make MRI data usable for image analysis. The first step was the intensity non-uniformity correction (Sled et al., 1998). These images were then linearly registered (aligned) into stereotaxic coordinates (Collins et al., 1994) based on the Talairach atlas (Talairach and Tournoux, 1988). The linear registration to Talairach coordinates was accomplished through 3D cross-correlation between a given volume and an average MR brain image previously converted into the Talairach coordinate system (Collins et al., 1994). After the registration the images had the same size and orientation, allowing for direct anatomical comparisons between subjects. A second non-uniformity correction was performed after the registration, which helped to remove any residual non-uniformity artifacts.

Every voxel in a non-uniformity corrected and registered image was then classified into one of the three classes: cerebro-spinal fluid (CSF), gray matter (GM), and WM using an automated tissue classification algorithm (Zijdenbos et al., 1998). Subsequently, cortical surface extraction from the tissue-classified images was performed, resulting in a 3D reconstruction of the cortical surface. Next, the skull and scalp were removed in the tissue-classified images using the 3D surface extraction as a mask in order to obtain the tissue volumes of the whole brain. Thus, the volumes of CSF, GM, and WM reported in this study were calculated using the tissue-classified images, which excluded the skull, scalp, cerebellum, and brainstem. The combination of the GM and WM was used as the whole brain volume in the VBR analysis.

The hippocampi were manually outlined using Analyze TM 8.1 (Brain Imaging Resource, Mayo Clinic, MN) by an experienced tracer (JM) from coronally orientated MR images in the anterior-posterior direction. Calculations of volumes were computed automatically by multiplying the number of voxels traced in each slice, by their depth (i.e., slice thickness). As described by Watson et al. (1992, 1997), the anterior tip of the HPC until the slice before the opening of the crux of the fornix was measured as the HPC head and body and included the subiculum, CA1-areas, and dentate gyrus. The HPC tail was measured from the slice immediately posterior to that which represented the last slice according to the Watson protocol (see Maller et al., 2007 for a more detailed description of this procedure) (Maller et al., 2007).

The CC was manually traced on the midline sagittal slice of the T1 images using anatomical landmarks in an hierarchical order on a Windows XP Professional workstation (Core2Duo CPU, 2GIG RAM) using the Region of Interest module within analyzetm 8.1 (Brain Imaging Resource, Mayo Clinic, MN, USA). The landmarks based on the midline sagittal slice were, first, no WM or only minimal WM in the cortical mantle surrounding the CC, second, the interthalamic adhesion, and third, the transparent septum and the visibility of the aqueduct of Sylvius. To adjust for total brain volume, total midsagittal CC area and every subregional area in the analyses data were normalized by dividing by each individual's total intracranial volume (ICV). ICV was calculated from the total of GM, WM, and cerebrospinal fluid volumes which were estimated from processing the T1-weighted scans through FSL 4.0 (Analysisgroup, 2012), using the FAST module.

#### Analyses

***Percentage of people showing significant decline in volume.*** As noted above, patients were classified as “decliners” if they showed atrophy in parenchymal tissue at a threshold of at least 2 z-scores below that of controls. (Note that we use the term “below” here rather than “greater than” for clarity of exposition.) We calculated a z-score for each patient participant using the following method: First, for each structure and substructure, percent change from time 1 to time 2 was calculated for patients and controls using the following formula: [t2 − t1/(t1 + t2)] * 100. Second, *monthly* percent change was then calculated in order to compare subjects with differing temporal gaps between scans. Third, a z-score was calculated for each patient using his/her *observed* score (i.e., percent change per month), the *expected* score (i.e., mean monthly percent change for controls), and the standard deviation for monthly percent change in the control group. We used a conservative z-score cut-off to classify decliners. Only if the patient was at least 2 z-scores below that of controls was he/she classified as a decliner. This enabled us to compute the percentage of decliners in our sample, in order to address the primary objective of our study.

***Relative vulnerability of decline of structures/sub-structures and pattern of deterioration.*** To examine whether structures were equally vulnerable to decline or whether the number of decliners varied across structures, we calculated 95% confidence intervals for each proportion of decliners across structures, and substructures. To examine whether decline in one structure was associated with decline in another, we calculated PHI coefficients and their 95% confidence intervals between structures and between substructures. This enabled us to measure the degree of overlap within subjects in decliner classification across the structures (i.e., is there a correlation between those classified as decliners and non-decliners in one structure and those in another?). The PHI coefficient is a measure of association for two binary variables, interpreted similarly to the Pearson Correlation Coefficient.

***Presentation of findings.*** Results are presented by each of the three research questions: (1) the overall percentage of people showing decline on at least one structure, and the number of structures on which subjects declined; (2) the relative vulnerability of structures (i.e., respective number of decliners in each structure), and (3) the pattern of atrophy (i.e., whether decline in one structure was associated with decline in another).

## Results

### Demographic comparisons between controls and patients

There was no significant difference between the patients and controls for age, *t* = 0.897 (*df* = 72), *p* = 0.373, Cohen's *d* = 0.211. The controls had significantly more education than the patients, *t* = −4.25 (*df* = 72), *p* < 0.001, Cohen's *d* = −1.00.

### Decliners: percentage of patients who show atrophy that is at least 2 z-scores below that of controls

Table 2 shows the monthly percent atrophy in the control group. The control group monthly percent changes were extremely small, with all scores close to 0, consistent with stability over time. [Note that while the primary aim of the study was to ascertain the *percentage* of decliners in our sample, we have included patient means with unpaired *t*-test results (all assuming unequal variances based on Levene's tests) to permit group mean comparisons]. These illustrate highly significant group differences.

**Table 2:** **Percent decline (by group) across structures and substructures**.

**Structure**	**Percent atrophy per month in healthy control patients (*N* = 12)**	**Percent atrophy per month in participants (*N* = 56)**
**VBR^a^**	0.18 (0.21)	1.32 (1.21)^*****^
HPC-L head	−0.008 (0.03)	−0.300 (0.63)^**^
HPC-L body	−0.004 (0.04)	−0.364 (0.59)^*****^
HPC-L tail	0.054 (0.08)	−0.676 (1.44)^*****^
**HPC-L total**	−0.002 (0.02)	−0.348 (0.48)^*****^
HPC-R head	0.004 (0.03)	−0.248 (0.60)^**^
HPC-R body	0.005 (0.03)	−0.331 (0.83)^**^
HPC-R tail	−0.023 (0.05)	−0.662 (1.05)^*****^
**HPC-R total**	0.002 (0.03)	−0.324 (0.53)^*****^
CC genu	−0.14 (0.11)	−0.855 (1.24)^*****^
CC body	0.09 (0.09)	−0.765 (1.33)^*****^
CC splenium	0.08 (0.09)	−0.805 (0.81)^*****^
**CC total**	−0.02 (0.04)	−0.812 (0.88)^*****^

a *Positive change for VBR denotes atrophy*.

** P < 0.005;

***** *P < 0.00001*.

Table 3 shows that compared to the normative group, over 96% of patients showed decline in at least one region. The majority (75%) showed decline in at least three of the four regions measured.

**Table 3:** **The number of patients who show atrophy**.

	**Number/(percentage) of decliners**
**Decline in at least 1 structure**	**54/56 (96.4%)**
Decline in 4/4 structures	22/56 (39.3%)
Decline in 3/4 structures	20/56 (35.7%)
Decline in 2/4 structures	8/56 (9%)
Decline in 1/4 structures	4/56 (7%)
Decline in 0/4 structures	2/56 (3.6%)

In Table 4 the absolute number and percentage of decliners by structure are presented. Over 70% of patients showed atrophy within each of the right and left HPC, the CC and the whole brain; moreover, the lower bounds of the confidence intervals for each of these values was greater than 50%. Therefore, the results strongly support our hypothesis that more than 50% of patients would show atrophy of the brain.

**Table 4:** **The number of patients who show decline in each structure and substructure**.

**Structure/substructure**	**Number/(percentage) of decliners by structure**	**Lower and upper 95% CI at 2 z-score < controls**
**VBR**	**45** (80.4)	67.6–89.9
HPC-L head	31 (55.4)	41.5–68.7
HPC-L body	24 (42.9)	29.7–56.8
HPC-L tail	32 (57.1)	43.2–70.3
**HPC-L total**	**40** (71.4)	57.8–82.7
HPC-R head	38 (67.9)	54.0–79.7
HPC-R body	39 (69.6)	55.9–81.2
HPC-R tail	36 (64.3)	50.4–76.6
**HPC-R total**	**41** (73.2)	59.7–84.2
CC genu	26 (46.4)^*^	33.0–60.3
CC body	42 (75.0)	61.6–85.6
CC splenium	42 (75.0)	61.6–85.6
**CC total**	**43 (76.8)**	63.6–87.0

### Relative vulnerability to decline of structures and sub-structures

Examining similarities and differences across structures in the number of decliners, Table 4 shows that, the highest absolute number of decliners is in VBR; this is not surprising as VBR is an index of total brain volume loss that subsumes all structures. However, none of the structures (including VBR) differed significantly from one another with respect to the number of decliners, with confidence intervals overlapping substantially. Within the substructures, it is interesting to note that only the genu of the CC was different from its respective sub-structures, with significantly fewer decliners than in either the body or the splenium. These results did not support our hypothesis that the hippocampus, and particularly the hippocampal head would show greatest vulnerability.

### Pattern of atrophy across structures

Examining whether atrophy across structures and sub-structures was related, we examined the association between decliners. If a patient showed atrophy across time on one structure, were they likely to also show atrophy on another structure? Table 5A shows the PHI coefficients and their 95% confidence intervals between regions measured. There was an overarching relationship, that is, significant overlap, between VBR with both the CC and the right (but not left) HPC. The right and left hippocampus also overlapped significantly. There was no relationship between the CC and the hippocampi. These results partially supported our hypotheses, namely a relationship between VBR and individual structures, and the absence of relationship between some (though not all) of the structures measured.

**Table 5A:** **PHI coefficients across the structures measured**.

**PHI coefficients (95% CI)**				
	**VBR**	**HPC-L total**	**HPC-R total**	**CC total**
**VBR**	–	*r* = −0.01 (*CI* = −0.28–0.25)	*r* = 0.31^*^ (*CI* = 0.05–0.53)	*r* = 0.37^*^ (*CI* = 0.12–0.57)
**HPC-L total**	–	–	*r* = 0.33^*^ (*CI* = 0.08–0.55)	*r* = 0.03 (*CI* = −0.24–0.29)
**HPC-R total**	–	–	–	*r* = 0.15 (*CI* = −0.12–0.39)

Table 5B presents the PHI coefficients and their 95% confidence intervals between substructures. Here, significant overlap was observed between the right hippocampus head and tail, and between the genu and body and the genu and splenium of the CC, findings which overall did not support our hypothesis.

**Table 5B:** **PHI coefficients for sub-structures of the left and right hippocampus and the corpus callosum**.

	**PHI coefficients (95% CI)**		
	**HPC-L head**	**HPC-L body**	**HPC-L tail**
**HPC-L head**	–	*r* = −0.02	*r* = 0.02
		(*CI* = −0.28–0.24)	(*CI* = −0.24–0.28)
**HPC-L body**	–	–	*r* = −0.05
			(*CI* = −0.31–0.21)
	**HPC-R head**	**HPC-R body**	**HPC-R tail**
**HPC-R head**	–	*r* = 0.13	*r* = 0.29^*^
		(*CI* = −0.14–0.38)	(*CI* = 0.02–0.51)
**HPC-R body**	–	–	*r* = 0.16
			(*CI* = −0.11–0.40)
	**CC genu**	**CC body**	**CC splenium**
**CC genu**	–	*r* = 0.29^*^	*r* = 0.37^*^
		(*CI* = 0.03–0.51)	(*CI* = 0.12–0.58)
**CC body**	–	–	*r* = 0.14
			(*CI* = −0.13–0.39)

## Discussion

The primary purpose of the current study was to gain a better understanding of the scale of atrophy in the chronic phase of TBI. Our cohort was a Canadian sample of patients with brain injuries ranging from complicated mild to severe. More than 96% of our sample showed atrophy over time in at least one region and the large majority showed atrophy in at least three of the four regions measured. Therefore, these findings indicate substantive atrophy—we employed a threshold of at least two z-scores below that of controls—across several structures. Given the relationship between sub-acute atrophy and behavioral and functional outcomes (Sidaros et al., 2008, 2009; Farbota et al., 2012), and between total brain volume loss and clinical impairment (Tate et al., 2011), these findings are clinically concerning, especially as brain atrophy is a known predictor of Alzheimer's Disease (Frisoni et al., 2010).

A secondary purpose of the study was to begin to characterize this chronic atrophy by examining two related features of the data: (i) the extent to which the number of decliners in each structure and sub-structure differed, allowing us to evaluate preliminarily the relative vulnerability of structures examined, and (ii) the extent to which decline in one structure was associated with decline in another. With regard to the first question, we found few differences in the number of decliners across structures and sub-structures, with only the genu differing significantly from the other sub-structures of the CC. Thus, contrary to our hypothesis, the findings in the small number of regions examined in our study do not suggest that one region is more vulnerable to chronic volume loss than another, with the exception of the genu. However, it is possible that limited power may have elevated Type II errors; given the rather large confidence intervals secondary to sample size, the results may under-represent differences between structures.

Regarding the second question, we found more associations across structures than we had predicted. Subjects showing atrophy in the whole brain also showed atrophy in other structures measured (i.e., CC and right HPC) as expected, which likely does not speak to underlying mechanisms, but rather to VBR's inclusive relationship with the other structures measured. However, we also found that those who declined in one HPC were more likely to also decline in the other. Within the sub-structures, those who declined in the right HPC head were more likely to decline in the tail, and those who declined in genu of the CC were more likely to decline in the body and the splenium. It is important to note that wide confidence intervals secondary to sample size bias the findings in favor of Type II errors, and that these findings may actually *underestimate* the degree of association between the structures.

These findings offer some direction for future research into mechanisms. A great deal of research has examined the *acute* primary and secondary mechanical, chemical, and electrophysiological changes in the brain following TBI, which in more serious injuries are ultimately associated with necrotic or apoptotic death (Povlishock and Katz, 2005; Griesbach et al., 2007). In contrast, there has been relatively limited research directly investigating mechanisms of later atrophy of the brain (but see Johnson et al., 2013; Smith et al., 2013).

### Mechanisms of atrophy in the chronic stage of TBI

In broad strokes, atrophy in the chronic stages of injury may reflect volume loss, neuronal death or a reduction in neuronal proliferation and/or survival. Cell death may occur due to delayed apoptotic mechanisms (Colicos et al., 1996; Bramlett et al., 1997a,b; Dixon et al., 1999; Williams et al., 2001; Coulson et al., 2008) secondary to trans-neuronal degeneration (Gennarelli and Graham, 1998; Tate and Bigler, 2000) arising from disconnections within or between functionally related structures (Tate and Bigler, 2000; Duering et al., 2012; Bigler, 2013b). Volume loss may reflect decreased complexity of the neuropil, with decreased spines, synapses and arborization or reduced fluid as a result of diminished protein production. Vascular changes might also contribute to volumetric changes over time. For example, reduced functional hyperemia (because of reduced neuronal demand) would reduce blood volume as would disrupted neurovascular coupling. A mechanism receiving increasing scientific attention for chronic stage losses in TBI is persisting inflammation, associated with cytokine release and microglial activation (Gentleman et al., 2004; Rodriguez-Paez et al., 2005; Johnson et al., 2013; Smith et al., 2013). Genetic causes of poorer clinical outcomes have been advanced for a number of years (Ponsford et al., 2011). A highly probable cause of volume losses are the synergistic effects of aging and TBI, as described in detail by Bigler (2013b); such effects may exacerbate the burden of normal and pathological aging, and thereby account for observed volume losses, with such losses also hastening the onset of dementia. Links between TBI and dementia are being increasingly made (Fakhran et al., 2013), with proteins implicated in dementias (e.g., amyloid-beta, amyloid precursor protein, tau), for example, found to accumulate in damaged axons and other neuronal compartments in the chronic stages of TBI (Bramlett et al., 1997b; Smith et al., 2003; Uryu et al., 2007; Johnson et al., 2012; Bennett et al., 2013).

Importantly, these candidate mechanisms of atrophy are empirically testable. Ultimately, an understanding is needed of whether a single mechanism or multiple mechanisms influence atrophy, whether different mechanisms affect different regions, and if multiple mechanisms do influence atrophy, whether they are additive or synergistic.

The findings in the current study favor some mechanisms over others, helping at least to constrain hypotheses for future research. The *ubiquity* of atrophy across patients would not support an exclusive genetic explanation of atrophy; for example, base rates of the e-4 allele that has been implicated in the relationship between TBI and dementia are considerably lower than the 96% incidence of patients who showed volume loss in this study.

With regard to neuroinflammation, Johnson et al. (2013) showed evidence of increasing neuroinflammation in the CC from the sub-acute to the more chronic stages of injury (i.e., 2 weeks to 1 year vs. > 1–18 years post-injury), and inflammation correlated with WM integrity losses and visible pathology. The prevalence of unequivocal markers of neuroinflammation appeared to be substantively lower than the 76.8% of patients in our study showing CC volume loss. However, neuroinflammation is likely one of multiple causes of volume loss in our study.

In humans, the identification of behavioral factors that may exacerbate or buffer against volume loss is of high importance given the potential for clinical intervention. It has been postulated that a downward spiral of negative neuroplastic change secondary to disuse may play a role in chronic decline (Evans, 2008; Miller et al., 2013). Supporting this putative mechanism, our group observed a significant negative correlation between self-reported hours of environmental enrichment in the first year post-injury and the degree of hippocampal atrophy observed from 5 to 20 months post-injury (Miller et al., 2013). Compounding disuse is physiological disconnection of healthy tissue from damaged tissue. Given that all of our patients had sustained brain damage and were therefore at risk of both disconnection and disuse, and moreover, that the CC and hippocampus are frequently affected acutely, and are associated with discrete cognitive functions, this explanation is consistent with the high prevalence of chronic atrophy observed. Within the substructures of the (right) hippocampus, the significant association observed is consistent with this interpretation, with the unique pattern of interconnectivity within the hippocampus meaning that damage to one area may deafferent another; if the disconnected tissue does not functionally re-organize, then it is vulnerable to transneuronal degeneration (McCarthy, 2003; Amaral et al., 2008).

However, other aspects of our findings do not support the interpretations above. For example, we found that hippocampal volume loss on the right was positively associated with volume loss on the left. Experience-dependent volume increases in the hippocampus (Draganski et al., 2006; Maguire et al., 2006) would have predicted that greater reliance on the less damaged hippocampus would result in volumetric increases to it, giving rise to a *negative* association between hippocampi. The positive association between hippocampi suggests a common mechanism deleteriously affecting both. One such mechanism is reduction in new neuronal growth, survival and integration. As is the case for many TBI patients, many of the patients in our cohort at 5 months post-injury and later, had residual physical impairments, reduced volition, had neither returned to work or school, were less socially engaged, and had limited access to resources (Frasca et al., 2013), Therefore, many underwent less physical activity, and many were engaged in less cognitively demanding activity. Since physical and cognitive enrichment have been associated, respectively, with enhanced neuronal proliferation and survivorship (Curlik and Shors, 2010, 2013), this reduced enrichment may have offset hippocampal growth. Moreover, widespread damage to networks might have further impeded integration of new neurons.

The array of possible interpretations for these findings indicates that much further research is needed to understand mechanisms of atrophy in sub-acute and chronic TBI. Such an understanding is critical for the development of treatment research to avert or abate this atrophy.

There were limitations of the current study. The size of the control group was relatively small and may have compromised the reliability of our findings. As well, because the timelines of the two assessments of controls and patients differed, we calculated monthly percent change to compare patients with controls. This calculation assumes a linear month-to-month change, which is not substantiated. There were significant education, but not age, differences between the patient and control groups. However, we speculate that this difference did not contribute to our findings. In a previous study by our group Miller et al. (2013) using overlapping participants, there was no relationship between years of education and degree of hippocampal atrophy. Moreover, the weight of evidence suggests that education confers protection against the clinical (cognitive) expression of disease, but not against the development of neuropathology or neurodegeneration itself (Members et al., 2010). As well, our findings do not permit us to distinguish between cell death versus volume loss without death. Other methodological approaches, including neuropathological ones, are needed to examine this distinction.

## Conclusions

In the chronic stage of moderate-severe atrophy, loss of volume is substantive and ubiquitous across patients. Changes may be attributable to tissue shrinkage—the result of lost neuropil, protein and/or fluids—or to cell death, with disconnection and disuse, inflammation and delayed apoptosis contributing independently or interactively. Environmental enrichment could play a role in offsetting these changes, and in the chronic stages of injury is a “no-harm” intervention that warrants investigation. Further research is needed to identify precise mechanisms of atrophy that would help us to develop targeted clinical interventions.

### Conflict of interest statement

The authors declare that the research was conducted in the absence of any commercial or financial relationships that could be construed as a potential conflict of interest.
